# Case report: A rare case of death due to end-stage renal disease caused by *Tripterygium wilfordii*-induced myelosuppression

**DOI:** 10.3389/fmed.2022.1036422

**Published:** 2022-11-30

**Authors:** Wen Zhang, Xinyin Liu, Cong Xia, Lingzhi He, Hongzhen Ma, Xiaoran Wang, Peipei Zhang

**Affiliations:** ^1^Department of Nephrology, The First Affiliated Hospital of Zhejiang Chinese Medical University (Zhejiang Provincial Hospital of Chinese Medicine), Hangzhou, China; ^2^The First Clinical Medical College, Zhejiang Chinese Medical University, Hangzhou, China; ^3^Department of Nephrology, The First People’s Hospital of Hangzhou Lin’an District, Hangzhou, China

**Keywords:** traditional Chinese medicine, *Tripterygium wilfordii*, myelosuppression, end-stage renal disease, case report

## Abstract

*Tripterygium wilfordii—*a traditional Chinese herbal medicine—is used to treat several diseases, including chronic kidney disease, rheumatic autoimmune disorder, and skin disorders. With the development of modern pharmacology, scientists have gradually realized that *T. wilfordii* has side effects on several organs and systems of the human body, including the liver, kidney, reproductive system, hematopoietic system, and immune system. Our understanding of its toxicity remains unclear. The incidence of problems in the hematopoietic system is not low but few related studies have been conducted. The serious consequences need to be of concern to clinicians and scientists. To ensure the safety of patients, it is important to elucidate the mechanism underlying the damage to the hematopoietic system caused by *T. wilfordii* and strategies to reduce its toxicity. Routine blood and biochemical tests should be conducted when administering *T. wilfordii*, and in case of any abnormality, the medication should be terminated in time along with a comprehensive symptomatic treatment. Herein, we report the case of a 50-year-old Chinese female with end-stage renal disease (ESRD) who developed severe bone marrow suppression after taking a short-term normal dose of a *T. wilfordii*-containing decoction. She died of sepsis and septic shock, although timely therapeutic measures (e.g., stimulating hematopoiesis, anti-infection treatment, and hemodialysis) were administered. To the best of our knowledge, this is the first report of death by *T. wilfordii*-induced myelosuppression from a short term, conventional dose in an adult female with ESRD. Although the underlying mechanism remains unclear, this case contradicts the notion that side effects on the hematopoietic system are non-lethal.

## Introduction

A 50-year-old female patient with end-stage renal disease (ESRD) who was not on renal replacement therapy took a *Tripterygium wilfordii*-containing decoction for 11 days, following which she developed obvious fatigue and scattered multiple subcutaneous ecchymoses on her lower limbs. The results of laboratory tests revealed abnormal coagulation function and peripheral hypocytosis mainly indicated by deceased leukocytes and platelets. Repeated bone marrow puncture results suggested acute suppression caused by medicinal ingredients. Later, the patient suffered a serious pulmonary infection. During her hospitalization, timely therapeutic measures were undertaken, including stopping the decoction usage, preventing bleeding, stimulating hematopoiesis, blood transfusion, anti-infection treatment, and hemodialysis. However, there was no change in the patient’s condition owing to persistent bone marrow suppression. Finally, she died of sepsis and septic shock after 2 months due to a serious infection.

To the best of our knowledge, this is the first report of death in an adult female patient with ESRD who developed severe bone marrow suppression after taking a short-term normal dose of *T. wilfordii*-containing decoction. Although the underlying mechanism remains unclear, it contradicts the notion that side effects on the hematopoietic system are non-lethal. The safety of administering *T. wilfordii* to patients with ESRD needs further evaluation, and a more detailed study on the mechanism of its toxic effects is essential.

## Case presentation

### First hospitalization

**FIGURE 1:**
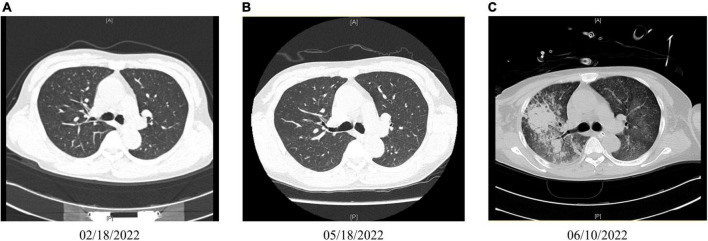
Chest imaging. **(A)** Normal computed tomography (CT) image taken on February 18, 2022. **(B)** Normal CT image taken on May 18, 2022. **(C)** The CT image taken on June 10, 2022 shows scattered large high-density shadows in both lungs.

On February 17, 2022, a 50-year-old Chinese female patient was admitted to Zhejiang University of Traditional Chinese Medicine First Affiliated Hospital in China with stage 5 chronic kidney disease, hypertension, renal anemia, and hyperuricemia. She had suffered from chronic kidney disease for more than 10 years, which developed into stage 5 approximately 2 years previously. Her renal pathological diagnosis was unclear. The patient was admitted for backache, nausea, and vomiting, and underwent a battery of routine tests (Table 1). Her body mass index (BMI) was 22.9. Physical examination was negative. Other examinations suggested that immunoglobulin G4, tumor markers, light chain test results, and thyroid function were normal. The antinuclear antibody spectrum showed a titer of 1:80; anti-Sjogren’s syndrome antigen A/Ro antibodies were positive, but the patient denied the relevant suspected clinical manifestations. Computed tomography (CT) of the chest showed normal images (Figure 1A). Emission computed tomography (ECT) of the kidneys revealed that the estimated renal plasma flow (left kidney: 13.08 ml/min; right kidney: 32.31 ml/min) and glomerular filtration (left kidney: 2.13 ml/min; right kidney: 2.42 ml/min) rates were low. We advised renal replacement therapy to the patient, but she refused it and asked for conservative treatment. We formulated the following treatment plan: compound α-ketoacid 3.78 g–3 times per day, roxadustat 120 mg–3 times per week, felodipine 5 mg–twice a day, calcium dobesilate 0.5 g–3 times per day, sodium bicarbonate 1 g–3 times per day, febuxostat 40 mg once daily, and beraprost sodium 40 μg–3 times per day. The patient was discharged, and her follow-up was scheduled as a nephrology outpatient.

**TABLE 1:** Laboratory data.

Variable	First hospitalization	Second hospitalization	Before death	Reference range
			
	02.18.2022	05.17.2022	06.17.2021	
**Blood**				
White blood cell count (per mm^3^)	3,600	2,600	110	3,500-9,500
Neutrophils (%)	66.0	77.7	21.3	40-75
Lymphocytes (%)	23.1	16.7	40.2	20-50
Neutrophils (per mm^3^)	2,400	2,000	10	1,800-6,310
Lymphocytes (per mm^3^)	800	400	10	1,100-3,210
Red blood cell count (per mm^3^)	2,660,000	2,320,000	2,020,000	3,800,000- 5,100,000
Hemoglobin (g/l)	58	67	58	130-175
Platelet count (per mm^3^)	487,000	21,000	5	125,000-350,000
Uric acid (μmol/l)	670	275	21	155-357
Creatinine (μmol/l)	171	299	80	45-84
Urea nitrogen (mmol/l)	10.76	36.1	11.9	2.6-7.5
Estimated glomerular filtration rate	13.02	15.31	77.92	
Total protein (g/l)	74.1	65.0	57.4	65.0-85.0
Albumin protein (g/l)	41.0	33.8	29.5	40.0-55.0
Potassium (mmol/l)	4.14	5.34	4.63	3.50-5.30
Sodium (mmol/l)	138.7	137.0	136.5	137.0-147.0
Chlorine (mmol/l)	104.2	103.2	101.7	99.0-110.0
Calcium (mmol/l)	2.27	2.20	2.30	2.10-2.60
Phosphorus (mmol/l)	1.51	1.66	0.42	0.81-1.65
Prothrombin time (s)	11.90	11.00	14.70	9.80-14.00
Prothrombin-time international normalized ratio	1.00	0.92	1.25	0.80-1.20
Fibrinogen (g/l)	4.18	3.83	5.22	2.00-4.00
Activated partial-thromboplastin time (s)	27.00	27.80	40.90	25.50-36.00
_*D*_-Dimer (mg/l)	1.09	1.52	8.29	0.00-0.55
C-reactive protein (mg/l)	<1.00	2.91	238.94	0.00-8.00
Erythrocyte sedimentation rate (mm/h)	–	24	–	0-20
Brain natriuretic peptide (ng/l)	80.7	–	1397.3	0.0-100.0
Cardiac troponin I (μg/l)	–	–	0.037	0.000-0.026
Procalcitonin (μg/l)	–	–	2.360	0.000-0.046
Parathormone (pmol/l)	10.88	19.1	–	1.59-6.89
**Arterial blood gas measurements**				
pH	7.392	7.401	7.370	7.350-7.450
Partial pressure of carbon dioxide (mmHg)	36.2	35.3	43.0	35.0-48.0
Partial pressure of oxygen (mmHg)	115.0	108.0	121.0	80.0-100.0
Standard bicarbonate (mmol/l)	22.3	22.3	24.90	22.0-28.0
Actual bicarbonate (mmol/l)	21.6	21.5	24.80	22.0-28.0
Standard base excess (mmol/l)	−2.6	−2.6	−0.40	−3.00-3.00
Actual base excess (mmol/l)	−2.5	−2.5	−0.30	−3.00-3.00
Lactic acid (mmol/l)	0.70	0.80	0.80	0.5-2.2
**Urine**				
Color	Yellow	Yellow	–	
Clarity	Clear	Clear	–	
Specific gravity	1.008	1.009	–	1.003-1.030
pH	6.0	7.0	–	4.5-8.0
Protein	2 +	2 +	–	
α1-Microglobulin (mg/l)	83.73	96.51	–	0.00-12.50
β2-Microglobulin (μg/l)	21.04	64.15	–	0.0-300.0
Microalbumin (mg/l)	995.9	669.7	–	0.0-30.0
Microalbumin/creatinine (mg/mgCr)	2.029	2.158	–	0.000-0.030
White blood cells (/μl)	6.0	5.6	–	0.0-9.0
Red blood cells (/μl)	22.2	22.2	–	0.0-13.0

Jaffe’s method was used to measure serum creatinine at our hospital. Before May 19, 2022, calcium dobesilate, which influences the detection of serum creatinine, was used to improve the patient’s microcirculation.

### Second hospitalization

**FIGURE 2:**
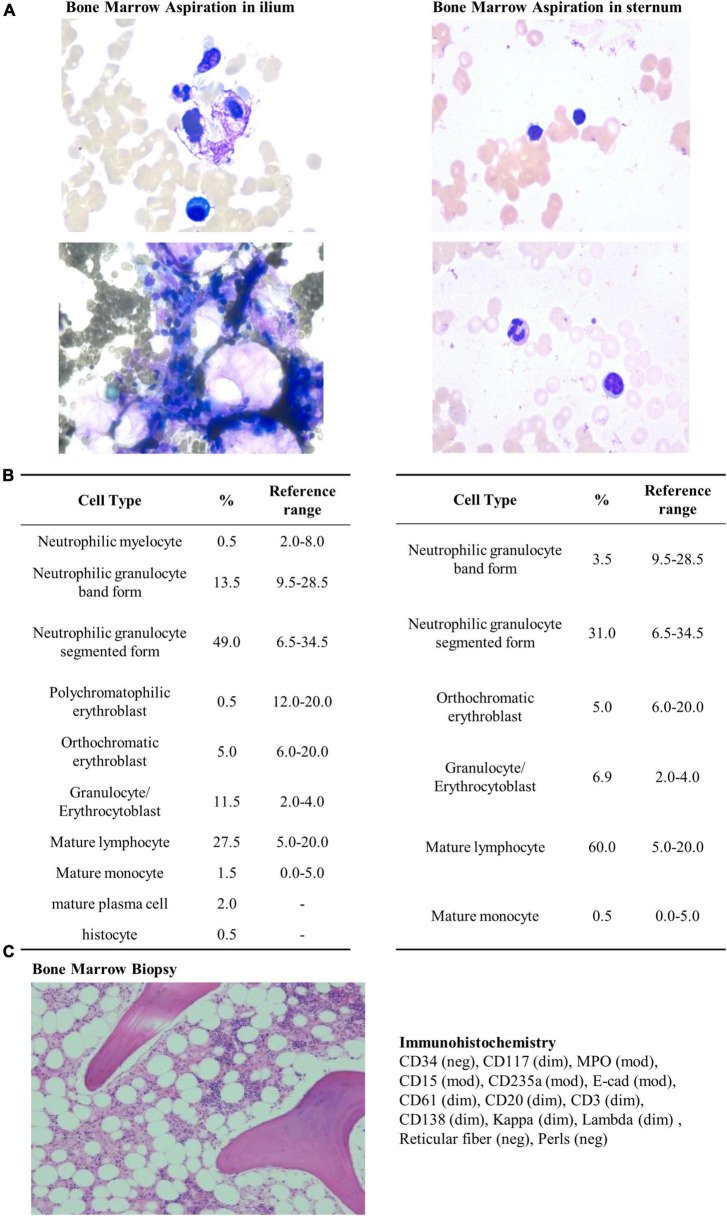
Bone marrow aspiration, biopsy, and immunohistochemistry. Bone marrow aspiration was performed using Wright’s staining technique. Bone marrow biopsy and immunohistochemistry were performed using staining techniques involving hematoxylin, Giemsa, acid fuchsin, reticular fiber, and Prussian blue stains. **(A)** Bone marrow aspiration in ilium performed on May 20, 2022. The results reveal low proliferation of nucleated cells without abnormality in morphology. **(B)** Bone marrow aspiration in sternum performed on May 24, 2022. The results reveal low proliferation of nucleated cells without abnormality in morphology. **(C)** Bone marrow biopsy and immunohistochemistry performed on May 20, 2022. Hematopoietic elements are substantially reduced (30%), and bone marrow space is replaced with adipose tissue (70%). Granulocyte and erythrocyte development are normal.

On May 04, a test of the patient’s urine confirmed persistent proteinuria, and the patient agreed to be prescribed *T. wilfordii*-containing decoction per day to preserve residual renal function, but she requested an active treatment plan, so the dosage of *T. wilfordii* was set as 12 g per day, which was the maximum dose within the safe range, for 14 days. The patient was required to decoct *T. wilfordii* for 2 h first. We advised the patient to consult a nephrologist if she experienced any discomfort, including fever, subcutaneous ecchymosis, nausea, and vomiting; if not, routine blood and biochemical tests should be conducted after 2 weeks. The patient gave informed consent. 11 days later, the patient showed obvious fatigue and subcutaneous ecchymoses for the first time and discontinued the Chinese medication. On May 17, she visited our hospital and underwent relevant examinations (Table 1). Her BMI did not change. Physical examination was negative except for multiple scattered subcutaneous ecchymoses on her lower limbs. ECT of the patient’s kidneys revealed a lower glomerular filtration rate (left kidney: 1.16 ml/min; right kidney: 2.52 ml/min) than before and estimated renal plasma flow was not detected. CT images of the chest were normal (Figure 1B). We admitted the patient to the intensive care unit. The patient showed obvious bone marrow suppression indicated by the deceased leukocytes and platelets, accompanied by abnormal coagulation function. However, she denied any previous hematopoietic system-related diseases. We prescribed dexamethasone, avatrombopag, recombinant human granulocyte colony-stimulating factor, recombinant human erythropoietin, and recombinant human thrombopoietin to stimulate hematopoiesis; carbazochrome sodium sulfonate to prevent bleeding; intravenous immunoglobulin to gain passive immunity; and repeated blood transfusion of red blood cells, albumin, human fibrinogen, platelets, and plasma. The patient consented to hemodialysis through a deep vein catheter. To clarify the cause of this condition, the hematology department was called upon for multidisciplinary combination therapy, and repeated bone marrow aspiration and biopsy were suggested (Figures 2A–C). The results showed that the patient’s hematopoietic functions were seriously inhibited. A Coombs test excluded autoimmune hemolytic anemia. Considering her history, we diagnosed acute bone marrow hematopoietic stagnation caused by drugs.

The therapeutic schedule of this patient remained unaltered before and after the appearance of abnormal hematopoietic function, except for the addition of *T. wilfordii* to the patient’s decoction. To the best of our knowledge, serious side effects related to the hematopoietic system have never been reported for the other herbal compounds in the decoction, based on “Buyang Huanwu Decoction” (1, 2), which was administered to the patient previously without any side effects on the hematopoietic system, for approximately 2 years. The composition formula is displayed in the Supplementary Table.

**FIGURE 3:**
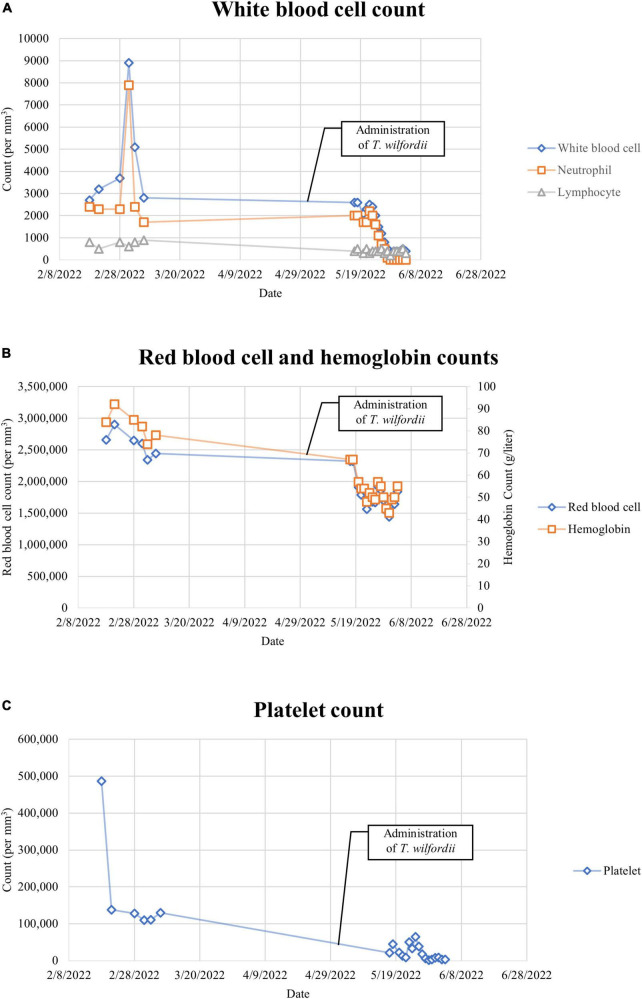
**(A)** White blood cell count. The normal reference ranges of the above data are listed in Table 1. **(B)** Red blood cell and hemoglobin counts. The normal reference ranges of the above data are listed in Table 1. **(C)** Platelet count. The normal reference ranges of the above data are listed in Table 1.

Combining with the results of blood routine tests before administering T. wilfordii, we believe that T. wilfordii caused bone marrow suppression (Figures 3A–C). Unfortunately, bone marrow suppression persisted throughout her second hospitalization. On May 31, the patient presented symptoms of dyspnea, cough, expectoration, and oxygen desaturation. CT images of the chest showed scattered large high-density shadows in both lungs, which suggested lung infection (Figure 1C). The results of sputum and blood culture suggested multidrug-resistant Enterobacter cloacae and carbapenem-resistant Acinetobacter Baumannii infection. Therefore, we administered several antibiotics including meropenem, polymyxin, tigecycline, cefoperazone sodium, and sulbactam sodium successively to treat the infection.

## Results

After more than 1 month of treatment, on June 18, the patient died of sepsis and septic shock.

## Discussion

### Efficacy of *Tripterygium wilfordii*

*Tripterygium wilfordii* Hook, belonging to Tripterygium of Celastraceae, has been used as a traditional Chinese medicine for hundreds of years. It is widely used to treat various diseases and shows remarkable curative effects. *T. wilfordii* is an antirheumatic Chinese medicinal herb. The earliest record that systematically summarized its efficacy in China is in “Ben Cao Gang Mu Shi Yi,” a book dating back nearly 300 years. It is used to treat various diseases including chronic renal disease, rheumatic immune disease, and skin disease (3). A meta-analysis of the treatment of chronic renal disease with *T. wilfordii* polycoride has shown that *T. wilfordii* can alleviate proteinuria and delay the progression of chronic renal disease (4).

Modern pharmacology suggests that *T. wilfordii* exerts anti-tumor, anti-inflammatory, and immunosuppressive effects (5, 6), and its main active ingredients are triptolide, celastrol, and total alkaloids of T. hypoglaucum (7). Its excellent curative effect is accompanied by some side effects; thus, scientists have conducted several studies to identify its toxic components and side effects and develop strategies to reduce them (8, 9). The toxic components and active components of T. wilfordii are largely overlapping. For example, triptolide, a diterpenoid epoxide in T. wilfordii, is a medicinal and noxious compound (7, 10).

### Methods of *Tripterygium wilfordii* drug delivery

At present, *T. wilfordii* is used clinically in two ways in China. One way is to use it in decoctions. Some studies have shown that combining it with other traditional Chinese medicines, such as licorice, silymarin, and ginseng, can help reduce the toxicity of *T. wilfordii* (11–13). The reference dose is different among Chinese pharmacopoeias and herbal guidelines. For example, the Traditional Chinese Pharmacology stipulated a dosage of 1–3 g of *T. wilfordii* per prescription in the formula and decoction time of 45–60 min before adding other herbs (14). In contrast, the Chinese Materia Medica recommended a dosage of 10–12 g of *T. wilfordii* per prescription in the formula and decoction time of 1–2 h (15). Our hospital suggests that the initial dosage of *T. wilfordii* should be different according to the patient’s weight. For adult patients weighing less than 60 kg, the dosage is 3 g per day; for those weighing more than or equal to 60 kg, it is 5 g per day. If there is no adverse reaction, the dose is increased to 12 g per day at most. The herb should be decocted for 2 h first. Considering that not all toxic substances have therapeutic effects, another method is to extract the effective components of *T. wilfordii* and convert them into a patented Chinese medicine to reduce toxicity. Among several preparations, *T. wilfordii* polycoride is the most convenient and widely used preparation, containing diterpene lactones, alkaloids, and triterpenoids (16). According to the Chinese Pharmacopoeia and National Standards published in 2010, the *T. wilfordii* lactone content should be not less than 0.1 mg/g/tablet; the recommended dose is 1.0–1.5 mg/kg/day, administered three times a day after meals (17).

In recent years, new methods of drug delivery have been proposed. Wang et al. attempted a transdermal microemulsion drug delivery system for *T. wilfordii* Hook f. to ameliorate its toxic effects on the male reproductive system (18). Xue et al. reported the protective effects of *Tripterygium* glycoside-loaded solid lipid nanoparticles against toxicity to the male reproductive system (19). However, these drug delivery methods neither demonstrate protection of the kidney or hematopoietic system nor are widely used for now.

### *Tripterygium wilfordii* toxicity

*Tripterygium wilfordii* affects various systems and organs, causing reproductive toxicity (20), liver damage (21), and kidney damage (22). Relevant studies have focused on these aspects (23–25). The toxicity of *T. wilfordii* is generally considered to be related to its dose and duration, and most of the side effects are reversible (26). However, *T. wilfordii* sometimes causes damage to the hematopoietic system, which usually manifests as leukopenia and aplastic anemia (27). The incidence of this side effect is lower than that of liver injury, but not uncommon, and its mechanism is unclear (4, 27, 28). According to Kusy et al., celastrol, an important component of *T. wilfordii*, specifically impairs the development of B cells and erythrocytes in the peripheral blood, bone marrow, spleen, and peritoneal cavity, but in mature lineages, the adverse effects are transient, as recovery is complete 4 weeks after the removal of the drug (29). Pyatt et al. suggested that *T. wilfordii* directly blocks the ability of very early multilineage as well as lineage-specific committed hematopoietic progenitor cells to form colonies in a dose-dependent way, which might be related to nuclear factor-kappa B signaling (30). These studies cannot fully explain the conditions found in this case.

Wu et al. (31), Feng et al. (32), and Liu et al. (33) reported several severe cases of bone marrow suppression caused by excessive doses or long-term use of *T. wilfordii*. The medicine was terminated in these cases and blood transfusion was performed to stimulate the hematopoietic system. The patients were eventually rescued and bone marrow suppression was eliminated.

### Case characteristics

To the best of our knowledge, this is the first report of death in an adult female patient with ESRD caused by severe bone marrow suppression after taking a short-term normal dose of a *T. wilfordii*-containing decoction. This finding contradicts the prevailing belief that side effects on the hematopoietic system are non-lethal. The patient had no previous hematopoietic system-related diseases, dosage of *T. wilfordii* decoction complied with the specifications, consumption duration was short, and medication was stopped immediately after symptoms were detected. Therefore, the cause of severe bone marrow suppression was unclear. Subsequent treatment continued for nearly 2 months; however, the patient did not recover and finally died of serious infection. It is unknown whether ESRD was involved in the occurrence of bone marrow suppression. From this case study, it is reasonable to conclude that *T. wilfordii* side effects are probably not only limited to toxic accumulation, but also related to the immune system, thereby triggering hematopoietic cell destruction. Moreover, we cannot exclude the possibility of rare idiosyncratic drug reactions (34). However, owing to the lack of relevant basic research, further studies are required to confirm these conjectures.

### Limitations

This case report has some limitations. First, owing to the complex ingredients of *T. wilfordii* decoction, we could not detect the blood concentration of *T. wilfordii*. Thus, it is difficult to directly confirm whether bone marrow suppression was caused by the toxic accumulation of *T. wilfordii*. Second, according to the recommendations of the Chinese Materia Medica and Traditional Chinese Pharmacology, *T. wilfordii* is toxic and needs to be decocted for a long time to reduce toxicity before adding other herbs (14, 15). Although the patient was instructed to decoct *T. wilfordii* for 2 h, we could not determine whether this was strictly performed. Finally, the reference dosage of *T. wilfordii* varies greatly among guidelines. We may not propose the most effective and safe reference dosage for patients with chronic kidney disease due to the lack of relevant study.

### Future research directions

Proposals for novel methods of drug delivery to alleviate *T. wilfordii* toxicity are essential. At present, the content of TWHF in *T. wilfordii* polyglycoside tablets varies among manufacturers (35). It is necessary to quantify blood drug concentration in clinical settings. Pharmacokinetic studies and safety evaluation of *T. wilfordii* should be continued. Genetic testing may verify whether the severe side effects of *T. wilfordii* are related to heredity. Detailed medication guidelines should be prepared for patients with liver or kidney damage, pregnant women, and the elderly population. The mechanisms underlying the toxic effects should be studied in detail to avoid bone marrow suppression, and a systematic treatment plan to prevent side effects should be prepared.

## Conclusion

Although *T. wilfordii* has been used for hundreds of years, our understanding of its toxic effects remains incomplete, and the mechanism remains unclear. In addition to reproductive toxicity and liver and kidney injuries, hematopoietic system problems are possible. These serious consequences deserve clinicians’ attention. When using *T. wilfordii*, the initial dose should be small and routine blood and biochemical tests should be conducted regularly. In case of abnormalities, the medicine should be stopped in time and symptomatic treatments should be provided. The safety of *T. wilfordii* in patients with ESRD requires detailed evaluation. Elucidating the mechanism of *T. wilfordii*-induced hematopoietic system damage and seeking new methods to reduce its toxicity are necessary for clinical applications.

## Data availability statement

The original contributions presented in this study are included in the article/Supplementary material, further inquiries can be directed to the corresponding author/s.

## Ethics statement

Written informed consent was obtained from the individual for the publication of any potentially identifiable images or data included in this article.

## Author contributions

WZ and XL researched data and wrote the manuscript. CX, LH, HM, XW, and PZ reviewed the manuscript. All authors contributed to the article and approved the submitted version.
